# Isolation, Identification, and Characterization of an Unknown Impurity in Lovastatin EP

**DOI:** 10.3797/scipharm.1305-04

**Published:** 2013-07-01

**Authors:** Chandrakant Belwal, Praveen Kumar Goyal, Anup Balte, Sandeep Kolhe, Kamlesh Chauhan, Ajay Singh Rawat, Anand Vardhan

**Affiliations:** Sterling Biotech Research Centre, Sterling Biotech Limited, Vadodara-391421, India.

**Keywords:** Lovastatin, Isolation, Identification, Characterization, Chromatography, Prep HPLC

## Abstract

An unknown impurity in the fermentation-based drug substance lovastatin at 0.52 RRT was observed invariably in all batches when analyzed by HPLC as per the PhEur monograph. This impurity was isolated from the impurity-enriched sample using reversed-phase preparative HPLC and characterized by using spectroscopic (PMR, CMR, MASS, and UV) techniques as the structurally-related compound Monacolin-X, having the molecular formula C_24_H_34_O_6_ and the chemical name 2-methyl-3-oxobutanoic acid 1,2,3,7,8,8a-hexahydro-3,7-dimethyl-8-[2-(tetrahydro-4-hydroxy-6-oxo-2*H*-pyran-2-yl)ethyl]-1-naphthalenyl ester.

## Introduction

**Fig. 1:**
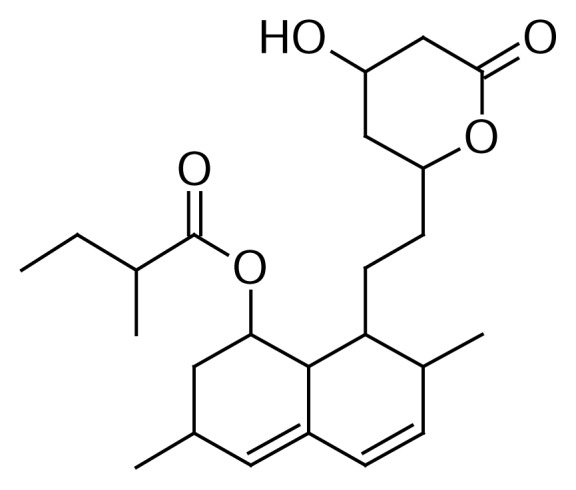
Structure of Lovastatin

Statins are widely used to lower the blood cholesterol level in patients of hyper-cholesterolemia. They competitively inhibit the rate-limiting enzyme of cholesterol biosynthesis, 3-hydroxy-3-methyl glutaryl coenzyme A (HMG-CoA) reductase [1]. Among the statins, mevastatin was the first to be investigated as a fungal secondary metabolite, later followed by lovastatin (monacolin K, Fig. 1) [2]. Lovastatin was the first statin approved by United States Food and Drug Administration as a hypocholesterolemic drug in August 1987 (FDA Orange Book Detail for application N019643 for approval for 20 mg tablets on Aug 31, 1987 and 40 mg tablets on Dec 14, 1988) [3]. Many microorganisms have been reported as lovastatin-producing fungi [4–6].

The purity of drugs is an important factor for the production of safe and effective pharmaceuticals. Most of the drugs obtained by fermentation process are purified either by a solvent extraction procedure or by chromatography. Although those procedures are used in most cases for their ease and fast purification, a chromatographic procedure has some advantage over solvent extraction procedures regarding the purity of drugs [7].

**Fig. 2:**
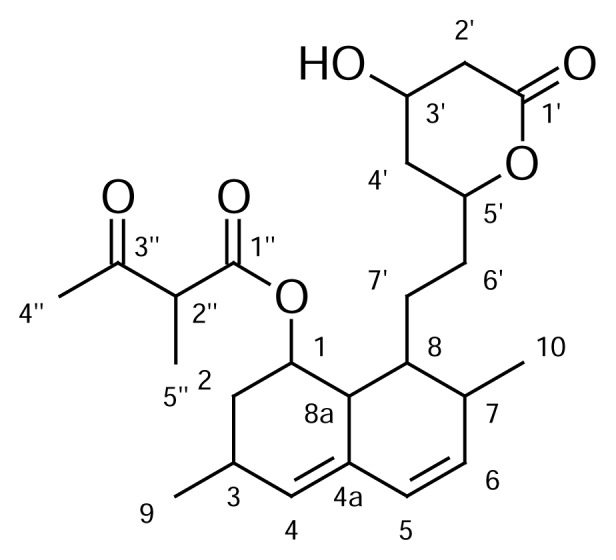
Structure of Lovastatin impurity at 0.52 RRT

During the routine analysis of lovastatin as per Ph. Eur., an impurity at 0.52 RRT, namely 8-[2-(4-hydroxy-6-oxotetrahydro-2*H*-pyran-2-yl)ethyl]-3,7-dimethyl-1,2,3,7,8,8a-hexahydro-naphthalen-1-yl 2-methyl-3-oxobutanoate (Fig. 2), was detected by an HPLC method [8]. A comprehensive study has been done to isolate and characterize this impurity by spectroscopic techniques. The requirement of identification and characterization of the impurity in the final drug substances is extremely necessary to meet the stringent regulatory or customer requirements [9].

## Materials and Methods

### Materials

The chemicals and reagents used for isolation and analysis are as follows: HPLC grade acetonitrile, Supplier: Spectrochem, India; Orthophosphoric acid, Supplier: S. D. Fine chem., India; Water: highly pure water using the Millipore Milli-Q Plus purification system; Lovastatin, source: Sterling Biotech Limited.

### Impurity Enrichment

An unknown impurity at the 0.52 RRT impurity was enriched to the 1.0% level, keeping the fact in mind that the 0.52 RRT impurity can be removed by crystallization in methanol, where the methanol mother liquor of lovastatin was repeatedly concentrated to remove lovastatin from it, and finally after 3–4 concentrations, ML was recovered completely to get the sample enriched (≈1% level) in the impurity.

### High-Performance Liquid Chromatography (Analytical)

A Waters Alliance separation module equipped with UV detector was used. LiChrospher RP Select-B column having dimensions 250 × 4.6 mm and 5 μm particle size was used for the analysis. The column was maintained at 35 °C, the eluent was monitored at 238 nm, and the data was recorded using Empower-II software. Mobile phase A (0.1% phosphoric acid in water) and mobile phase B (acetonitrile) were used for the separation in a gradient system with a flow rate of 1.5 mL/min (Table 1). The test solution was prepared by dissolving a 20 mg sample in 50 mL of acetonitrile. All parameters are shown in Table 1.

### High-Performance Liquid Chromatography (Preparative)

A Waters data prep Separation Module equipped with a 2487 UV detector and system controller was used. A combination of two columns, namely the Inertsil ODS-2 column having dimensions 250 × 50 mm, 10 μm particle size and Xbridge 30 mm × 50 mm, 5 μm particle size, were used for the impurity isolation work. A 10 mL injection loop was used, the eluent was monitored at 238 nm, and the data was recorded using Millenium software. About 1000 mg of the sample was dissolved in acetonitrile and loaded on the preparative column. A mixture of water and acetonitrile was used as the mobile phase as shown in Table 2. Flow rate was adjusted to 10 mL/min and the eluent was monitored at 238 nm.

### Mass Spectrometry

Mass spectra were obtained using the Waters Q-tof LC/MS-MS mass spectrometer in positive ion ionization mode.

### NMR Spectroscopy

Proton and carbon NMR measurements were performed on a Bruker Avance 500 MHz instrument at 25 °C in deuterated dimethylsulfoxide and chloroform and the chemical shift values were reported on the δ scale relative to TMS.

## Results and Discussion

### Detection of Impurities

**Fig. 3:**
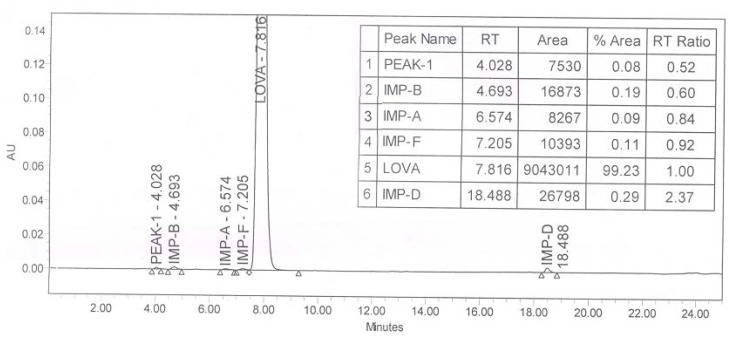
A typical HPLC chromatogram of lovastatin showing impurity at 0.52 RRT

A typical analytical HPLC chromatogram (Fig. 3) of lovastatin was recorded using an HPLC method described in the Ph Eur. monograph. The target impurity under study eluted at a retention time 4.02 min, and lovastatin eluted at 7.8 min. The target impurity (at 0.52 RRT) was isolated from the enriched impurity sample of lovastatin on the preparative HPLC.

### Isolation of Impurities by Preparative HPLC

A reversed-phase solvent system was used for the isolation of impurities. The enriched impurity sample was loaded on the preparative column and the pure fraction collected were combined together and analyzed using analytical HPLC to confirm the RRT and purity of the isolated impurity. The combined fraction was concentrated under high vacuum to distill out the solvent (acetonitrile). The remaining aqueous part was subjected to lyophilizer to get a pure compound. The chromatographic purity of the impurity was tested by analytical HPLC separately before and after concentration. The isolated solid impurity was subjected to spectral analysis.

### Structure Determination of Impurity

#### Mass Spectroscopy

**Fig. 4:**
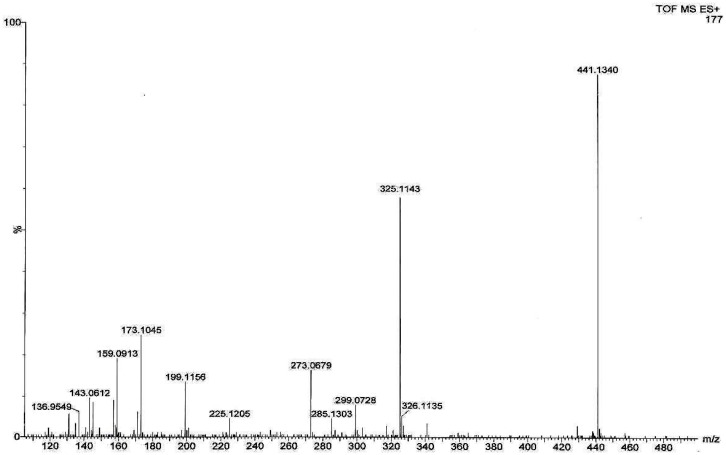
Mass spectrum of the isolated impurity

The mass spectrum of the impurity at 0.52 RRT in positive ionization mode (Fig. 4) exhibits an M+Na^+^ peak at m/z 441.13 atomic mass unit (amu) which corresponds to the exact calculated molar mass of Monacolin-X (418.5231), and the other fragment ions are at m/z 325.11, 299.07, 285.13, 273.06, 225.12, 199.11, 173.10, 159.09, 143.06, and 136.95.

The mass spectrum of the impurity at 0.52 RRT was compared to the mass spectrum of lovastatin and it was observed that the fragmentation pattern of the impurity is the same as the fragmentation pattern of the lovastatin, except for the molecular ion peak or sodium adduct peak (M+Na 441.13), indicating that the isolated impurity is structurally similar to lovastatin.

#### ^1^H-NMR Spectroscopy

The proton NMR spectrum of the impurity at 0.52 RRT in CDCl_3_ was taken. The NMR measurements were performed on a Bruker Avance 500 MHz instrument at 25 °C in deuterated chloroform and the chemical shift values were reported on the δ scale relative to TMS. The

**Fig. 5:**
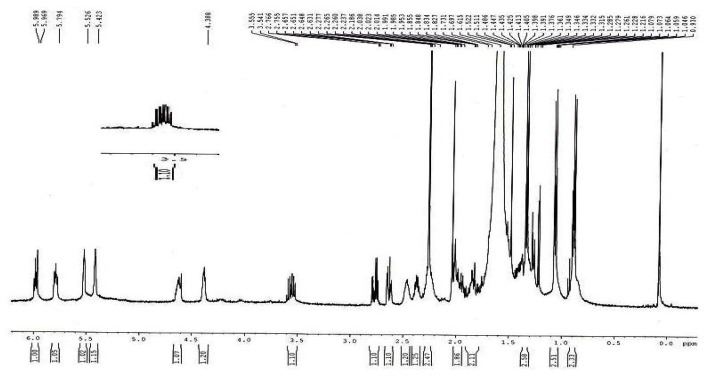
^1^H-NMR spectrum of the isolated impurity

^1^H-NMR spectrum of the isolated impurity (Fig. 5) was compared with lovastatin and found that it is structurally close to lovastatin except for some structural variations. The ^1^H-NMR spectrum of the isolated impurity suggests the presence of one –COCH_3_ group and one –COCH(CH_3_)CO- group; the presence of these groups can be confirmed by locating a singlet of three protons at 2.27 ppm and a multiplet of one proton at 3.54–3.55ppm as illustrated in Table 3.

#### ^13^C-NMR Spectoscopy

**Fig. 6:**
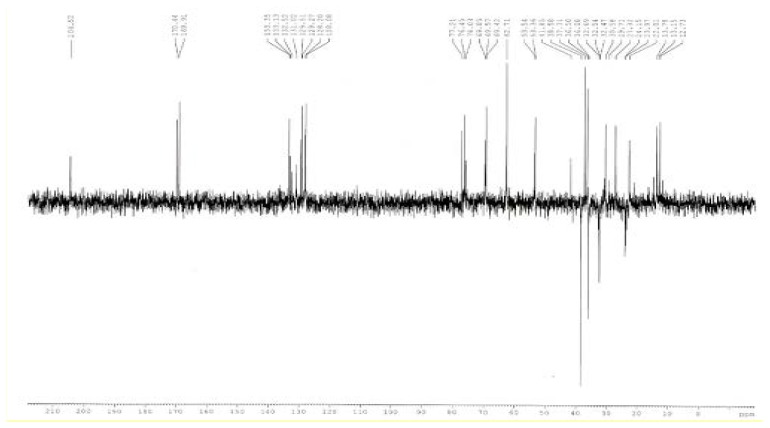
^13^C-NMR DEPT-135 spectrum of the isolated impurity

**Fig. 7:**
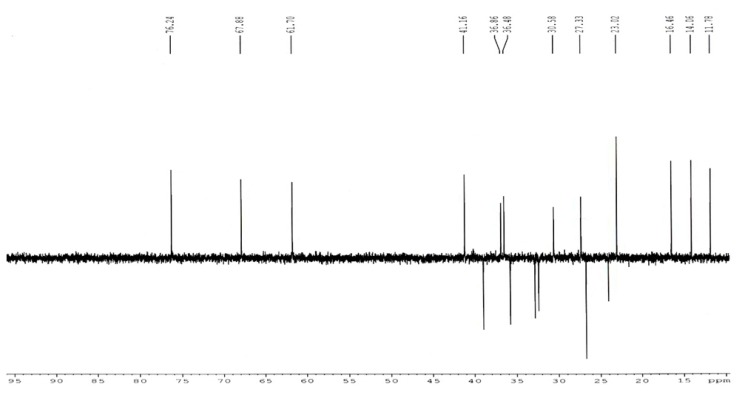
^13^C-NMR DEPT-135 spectrum of lovastatin

The carbon NMR spectrum of the impurity at 0.52 RRT in CDCl_3_ was taken. The NMR measurements were performed on a Bruker Advance 500 MHz instrument in deuterated chloroform and the chemical shift values were reported on the d scale relative to TMS. The ^13^C-NMR spectrum of the isolated impurity (Fig. 6) was further confirmed by comparing ^13^C-NMR signals with those of lovastatin (Fig. 7). The methylene signal at 26.75 ppm of C-3″ in lovastatin disappeared and a carbonyl signal at 204.52 ppm appeared in the isolated impurity, indicating a carbonyl group at C-3″. Similarly, the methyl signal at 11.78 ppm of C-4″ in lovastatin disappeared and an acetoxymethyl signal at 29.71 ppm appeared in the ^13^C- NMR spectrum of the isolated impurity.

The ^13^C-NMR DEPT-135 spectrum of the isolated impurity (Fig. 6) was further studied which confirmed the absence of a -CH_2_CH_3_ group at C-3″ which is –COCH_3_ in the case of the isolated impurity. The –CH_2_ signal at 26.75 ppm of C-3″ in lovastatin (Figure 5) shifted to a –COCH_3_ signal at 204.52 ppm in the isolated impurity indicating that a -CH_2_CH_3_ group changed to a –COCH_3_ group at C-3″. All the data are summarize in Table 4.

#### UV-Spectroscopy

**Fig. 8:**
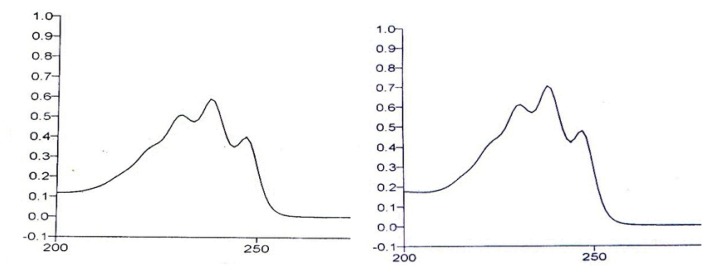
UV spectrum of the isolated impurity (left) and lovastatin (right)

The UV spectrum of the impurity at 0.52 RRT and lovastatin were measured on a Perkin Elmer instrument in acetonitrile and the maxima were observed at 238.2 nm for both lovastatin and the isolated impurity (Fig. 8).

## Conclusion

The mass spectroscopy data of the isolated impurity and lovastatin were compared and it was found that the fragmentation pattern of both the compounds are same except for the molecular ion peak or sodium adduct peak, indicating that the isolated impurity is structurally close to lovastatin. The UV spectrum of the impurity also indicates that the impurity is a related compound of lovastatin. The ^1^H-NMR spectrum of the isolated impurity suggests the presence of one –COCH_3_ group and one –COCH(CH_3_)CO- group. The presence of these groups can be confirmed by locating a singlet of three protons at 2.27 ppm and a multiplet of one proton at 3.54–3.55 ppm (Figure 3). The ^13^C-NMR spectrum of the isolated impurity confirms that the methylene signal at 26.75 ppm of C-3″ in lovastatin disappeared and a carbonyl signal at 204.52 ppm appeared in the isolated impurity, indicating a carbonyl group at C-3″. Similarly, the methyl signal at 11.78 ppm of C-4″ in lovastatin disappeared and an acetoxymethyl signal at 29.71 ppm appeared in the ^13^C-NMR spectrum of the isolated impurity. The ^13^C-NMR DEPT-135 spectrum of the impurity confirmed that the –CH_2_ signal at 26.75 ppm of C-3″ in lovastatin shifted to a –COCH_3_ signal at 204.52 ppm in the isolated impurity, indicating that a -CH_2_CH_3_ group changed to a –COCH_3_ group at C-3″. The above spectroscopic data suggest that the unknown impurity at 0.52 RRT in lovastatin EP is Monacolin-X [9], having molecular formula C_24_H_34_O_6_ and chemical name 2-methyl-3-oxobutanoic acid 1,2,3,7,8,8a-hexa-hydro-3,7-dimethyl-8-[2-(tetrahydro-4-hydroxy-6-oxo-2*H*-pyran-2-yl)ethyl]-1-naphthalenyl ester.

## Figures and Tables

**Tab. 1:** Analytical HPLC parameters

System: Waters Alliance High performance liquid chromatography (HPLC)		

Column: Lichrospher RP Select-B column having dimensions 250 × 4.6 mm		

Stationary Phase: Octylsilyl silica gel for chromatography 5 μm particle size		

Mobile phase: (A) 0.1% phosphoric acid in water, (B) acetonitrile		

Gradient program		

Time (min.)	Mobile phase-A (%v/v)	Mobile phase-B (%v/v)
0–7	40	60
7–9	35→40	65→60
9–15	10→35	90→65
15–20	10	90

Flow rate: 1.5 mL/min

Detection: Spectrophotometer at 238nm

**Tab. 2:** Preparative HPLC parameters

System: Waters data prep Separation Module equipped with 2487 UV detector
Column: Inertsil ODS-2 column 250 × 50 mm and Xbridge 30 mm × 50 mm
Stationary Phase: Octydecylsilyl silica gel for chromatography 10 μm and 5 μm, respectively
Mobile phase: Mixture of water and acetonitrile (50:50)
Flow rate: 10 mL/min
Detection: Spectrophotometer at 238nm

**Tab. 3:** Comparison of critical peaks in the ^1^H-NMR spectrum

Assignments	Chemical shift in δ ppm	

	Lovastatin	Isolated impurity
m, 1H (C-2″)	2.29–2.30	3.54–3.55
m, 2H (C-3″)	1.60–1.62	No peak
s, 3H (-COCH_3_) (C-4″)	No peak	2.27
t, 3H (-CH_2_CH_3_) (C-4″, J=7.8Hz)	0.81	No peak
d, 3H (-CH CH_3_) (C-5″)	0.84	0.93

**Tab. 4:** Comparison of peaks in ^13^C-NMR spectrum

Carbon number	Chemical shift in δ ppm	

	Lovastatin	Isolated impurity
1	69.68	69.57
2 (CH_2_)	32.42	32.55
3	27.35	27.33
3-CH_3_	23.04	22.86
4	129.61	129.28
4a	130.80	131.58
5	129.77	129.51
6	133.48	133.35
7	30.60	30.63
7-CH_3_	14.08	14.89
8	36.50	36.51
8a	36.90	37.30
1′	170.56	170.44
2′ (CH_2_)	39.00	38.70
3′	61.72	62.70
4′ (CH_2_)	35.89	36.09
5′	76.20	76.76
6′ (CH_2_)	32.86	32.69
7′ (CH_2_)	24.08	24.15
1″	175.93	169.91
2″	41.15	53.37
2″-CH_3_	16.46	12.74
3″	26.75 (CH_2_)	204.52 (C=O)
4″	11.78 (CH_2_–CH_3_)	29.71 (C=O-CH_3_)
